# No evidence for cardiotoxicity of miltefosine – Reply^⋆^

**DOI:** 10.1016/j.abd.2022.03.001

**Published:** 2022-06-03

**Authors:** Daniel Holanda Barroso, Ciro Martins Gomes, Antônia Marilene da Silva, Raimunda Nonata Ribeiro Sampaio

**Affiliations:** a Faculty of Medicine, Universidade de Brasília, Brasília, DF, Brazil; b Dermatomycology Laboratory, Faculty of Medicine, Universidade de Brasília, Brasília, DF, Brazil; c Tropical Medicine Center, Universidade de Brasília, Brasília, DF, Brazil; d Hospital Universitário de Brasília, Universidade de Brasília, Brasília, DF, Brazil

Dear Editor,

The authors are pleased to read Professor Dorlos’ comments on the article that we published in this journal.^1^ As the professor rightly states cardiotoxicity of Meglumine Antimoniate (NMG) is well established, and there is no convincing pharmacokinetic evidence that miltefosine causes cardiotoxicity. We, however, cannot arrive at any specific conclusion on the pharmacokinetics of miltefosine based on our data as we have not investigated drug absorption, distribution, metabolism, or excretion in this study. Also, many explanations are possible, other than miltefosine-specific cardiotoxicity, to account for the increase in QTc interval observed in Sundar’s et al. study.^2^ The present study suggests that miltefosine may have some specific but transitory cardiotoxicity since the same increase in the QTc interval was not observed on the meglumine antimoniate treatment during the same period. Certainly, as we have stated in the paper, our conclusions are exploratory and deserve to be confirmed by evidence from other studies. The actual absence of pharmacokinetic evidence to support our findings, however, does not exclude their relevance. As correctly stated by the author, the strategy employed in the study has to be viewed with skepticism, especially considering the increased type 1 error rate related to multiple comparisons.^3^ Nevertheless, the statistical analysis must be adapted to the characteristics of the variables studied. As precisely said, the QTc interval is not a clinical outcome, and its significance is related to the possibility of cardiac arrhythmias. To account for that, we have dichotomized the variable to separate patients with increased risk of arrhythmia (QTc ≥440 ms) and those with no increased risk (QTc <440 ms). Additionally, aggregating the different time points of the subjects may overlook a transitory and potentially fatal increased risk of cardiac death. Thus, we understand that the analysis in each time point is important to account for intrasubject variability in this case. The cardiotoxicity of miltefosine is not well established in the literature, and we continue to prefer this drug over NMG in patients with high cardiac risk. Nevertheless, any signal of miltefosine cardiotoxicity deserves to be reported, especially considering the increased interest of the scientific community in combination therapy, as evidenced by the many studies done on the subject.^4^, ^5^ Finally, considering that safety is the primary concern when dealing with human lives, any case of cardiotoxicity related to miltefosine should be included in the pharmacovigilance data. This is even more important now, once miltefosine has been incorporated in countries where leishmaniasis is endemic.

## Financial support

To the Brazilian Society of dermatology thought FUNADERM grant and Fundação de Apoio à Pesquisa do Distrito Federal (FAP-DF) grant number 0193.001447/2016 for their financial support.

## Authors’ contributions

Daniel Holanda Barroso: Drafting and editing of the manuscript; critical review of the manuscript.

Ciro Martins Gomes: Drafting and editing of the manuscript; critical review of the manuscript.

Antônia Marilene da Silva: Drafting and editing of the manuscript; critical review of the manuscript.

Raimunda Nonata Ribeiro Sampaio: Drafting and editing of the manuscript; critical review of the manuscript.

## Conflicts of interest

None declared.
